# Development of a cross-cultural HPV community engagement model within Scotland

**DOI:** 10.1177/0017896916685592

**Published:** 2017-01-23

**Authors:** Elaine Carnegie, Anne Whittaker, Carol Gray Brunton, Rhona Hogg, Catriona Kennedy, Shona Hilton, Seeromanie Harding, Kevin G Pollock, Janette Pow

**Affiliations:** a School of Health & Social Care, Edinburgh Napier University, Sighthill Campus, Edinburgh, UK; b NHS Greater Glasgow and Clyde, Glasgow, UK; c School of Nursing and Midwifery, Robert Gordon University, Aberdeen, UK; d MRC/CSO Social and Public Health Sciences Unit, University of Glasgow, Glasgow, UK; e Division of Diabetes & Nutritional Sciences, King’s College London, London, UK; f Vaccine Preventable Diseases, Health Protection Scotland, Glasgow, UK

**Keywords:** Community engagement, ethnic minorities, HPV, qualitative research, vaccination, young people

## Abstract

**Objective:**

To examine cultural barriers and participant solutions regarding acceptance and uptake of the human papillomavirus (HPV) vaccine from the perspective of Black African, White-Caribbean, Arab, Indian, Bangladeshi and Pakistani young people.

**Methods:**

In total, 40 young people from minority ethnic communities in Scotland took part in a qualitative study, involving seven focus groups and four paired interviews, to explore their views and experiences of the HPV vaccine. Using critical discursive psychology, the analysis focused on young people’s accounts of barriers and enablers to information, access and uptake of the HPV vaccination programme.

**Results:**

Participants suggested innovative strategies to tackle intergenerational concerns, information design and accessibility, and public health communications across diverse contexts. A cross-cultural community engagement model was developed, embracing diversity and contradiction across different ethnic groups. This included four inter-related strategies: providing targeted and flexible information for young people, vaccine provision across the life-course, intergenerational information and specific cross-cultural communications.

**Conclusion:**

This is the first HPV cross-cultural model inductively derived from accounts of young people from different ethnic communities. We recommend public health practitioners and policymakers consider using the processes and strategies within this model to increase dialogue around public engagement, awareness and receptivity towards HPV vaccination.

## Introduction

Human papillomavirus (HPV) is the most prevalent sexually transmitted infection among young people (Audisio et al., 2015). Three vaccines have been developed to prevent HPV-related diseases, targeting oncogenic and non-oncogenic genotypes. By 2014, 58 countries had introduced one of the vaccines into their national immunisation programme (World Health Organization [WHO], 2014a), with varying uptake rates reported (Smith et al., 2015).

Since 2008, the UK immunisation programme has offered girls aged 9–14 years protection against two oncogenic types – HPV16 and HPV18 – which are responsible for 70% of cervical cancer cases. More recently, a quadrivalent vaccine has been introduced, which also provides protection against HPV6 and HPV11, which cause anogenital warts (Immunisation Scotland, 2015). In Scotland, high vaccination rates have been reported (>90% uptake of full immunisation), although these obscure diverse understandings of HPV and intentions within multi-cultural groups (Sinka et al., 2014). More widely, the vaccine has been accepted for use among men in several countries, such as Australia and Canada, because it can prevent genital warts and oral, penile and anal cancers (Mirghani et al., 2014). In 2015, the UK Joint Committee for Vaccination and Immunisation recommended that genito-urinary clinics offer the vaccine to men who have sex with men (Department of Health, 2015). The debate about whether to offer the vaccine to all men in the UK remains contentious (Baker, 2014).

HPV vaccination programmes are delivered within varying contexts, such as schools and primary care (WHO, 2014b). Interventions to influence knowledge and intention have been developed with parents and young people from particular demographic groups based on ethnicity, migration and income (Fisher et al., 2013). However, conflicting evidence exists regarding vaccine uptake and levels of HPV-related disease awareness. Low levels of knowledge have been associated with low and high acceptability (Marlow et al., 2009), indicating that knowledge and education do not always lead to vaccination uptake but are important for informed decision-making. Within school-based programmes, acceptability may relate to abdication of responsibility by parents to state authority and provision (Lee Mortensen et al., 2015). One pan-European study (Lee Mortensen et al., 2015: 8) identified two major factors influencing vaccination uptake – knowledge and structural incentives: ‘When structural barriers are low, as in Italy and the UK, acceptability is high but knowledge does not necessarily follow. When structural barriers are higher, as in France and Germany, the need for knowledge and reassurance is higher’. Having investigated men’s decision-making, Wheldon et al. (2015) recommend further research to determine how perceptions of ‘severity’ or ‘immediacy’ of HPV-related morbidity, such as cancer and genital warts, can influence intentions or vaccine uptake.

Evidence also reveals that knowledge and acceptability of the vaccine are lower in some ethnic minority groups (Batista Ferrer et al., 2015; Roberts et al., 2011). Meanings of the term ‘ethnicity’ include shared origins, culture, language or traditions that are distinctive, maintained between generations and lead to a sense of identity and group (Senior and Bhopal, 1994: 327).

During a catch-up campaign in England, over six hundred 16-year-old girls from a further education college were recruited into a prospective study. Ethnicity was independently associated with vaccine uptake. Those from Black or Other ethnic backgrounds were less likely to have been vaccinated than those from White backgrounds (Bowyer et al., 2014). Using a cross-sectional design, Marlow et al. (2009) assessed HPV awareness and vaccine acceptability in a representative sample of women from the major UK minority ethnic groups. Minority ethnic women reported lower awareness than White women, regardless of the language spoken at home or whether they were UK- or foreign-born. Knowledge and intentions among young men from minority ethnic backgrounds within the UK are largely undocumented, although some evidence suggests that acceptability of the vaccine could be very low (Prue and Santin, 2015).

### Rationale for a community engagement model

Community engagement is one important facet of any health promotion approach that attempts to address health inequalities (Curtis, 2004: 278). Thompson (2003) frames empowerment within ‘a strategy for promoting equality’ (p. 220). Personal empowerment can challenge and undermine discrimination within linguistic, cultural and structural dimensions of power. Discursive psychology has been useful for critically examining these dimensions and strategies for promoting equality (Askew, 2016).

Misunderstandings and lack of knowledge regarding HPV vaccination have been associated with poor health literacy (Hunter and Weinstein, 2016; Watson and Serrant-Green, 2012). Promoting health literacy means going beyond the provision of high-quality information to designing and delivering health services in a way that meets the needs of diverse communities (NHS Scotland, 2014). Interventions developed with communities from the outset help ensure acceptability and effectiveness (Israel et al., 2008). Community engagement is one important facet of any approach that attempts to address health inequalities (Curtis, 2004: 278).

Adopting a bottom-up approach can inform the design and delivery of effective communication strategies, providing downstream benefit to improved public health (Dwyer-White et al., 2015). The development of a model that offers a sustainable approach to raising the profile of HPV within diverse communities could build on previous community engagement models such as identity-based, asset-based and community democracy models (Hashagen, 2002). These models emphasise process, whereby communities identify problems and solutions, which in turn fosters empowerment.

National HPV vaccination programmes tend to adopt universal delivery strategies rather than targeted cultural and demographic strategies. Thus, relational, institutional, cultural and contextual factors that shape sexual health practices are often ignored. Qualitative methodologies, such as discursive psychology, could address this gap, where issues of intersectionality and participation are key. This study examined the accounts of young people from Black, Asian and Minority Ethnic (BAME) communities in order to examine understandings and explanations for HPV-related health behaviours within differing cultural contexts. Drawing on inductively produced participant solutions, we outline a cross-cultural community engagement model for HPV which encompasses diversity across a range of BAME populations.

## Methods

### Design

The study involved seven focus groups and four paired interviews with a purposive sample of 40 young people from BAME communities, recruited from three Scottish geographical regions between June and November 2015. Underpinned by a critical public health paradigm and utilising critical discursive psychology (Potter, 1996; Willig, 2008), we explored how the young people talked about HPV and the vaccine. Critical discursive psychology attempts to examine and explain people’s accounts within a socio-cultural context in recognition that beliefs and behaviours are constituted within shared discursive and social practices (Willig, 2008).

Analysis centred on the ways in which the young people used interaction, language and different discourses to construct their opinions, identities and social worlds, as well as cultural barriers and culturally sensitive solutions regarding access and inclusion within the HPV vaccination programme.

### Participants

Over 80 community organisations and networks were contacted to invite their members (aged 16–26 years) to participate in the study. In total, 40 participants (28 women, 12 men) took part in the study. This number was large enough to include a diverse range of participants with differing characteristics, yet small enough to allow for an in-depth analysis of the data. Of the 40 participants, 24 were aged 16–19 years and 16 were aged 20–26 years. Participants lived in urban, suburban and semi-rural areas. In all, 28 described themselves as being of South Asian descent, 6 of Arab descent and 6 of Black African descent. Religious beliefs and practices were important to the majority of participants: Muslim (*n* = 21), Sikh (*n* = 12), and Christian (*n* = 3). Three were married and 12 stated they had been in a sexual relationship; 18 described themselves as students, 9 were employed, 8 unemployed and 5 still at school; and 23 of the women had received the vaccine.

### Procedure

The Research Integrity Committee of Edinburgh Napier University granted ethical approval to conduct the study. The first named author (E.C.) established links with ethnic community groups, societies and networks, who promoted the study and assisted with recruitment. Prospective participants were given a study participant information sheet that provided assurances about confidentiality and anonymity, and written informed consent was obtained.

Participants were invited to a gender-specific focus group or paired interview where they could feel comfortable and engage in shared activities, dialogue and debate (Barbour, 2008). The research setting was negotiated with the young people and included religious complexes and third sector offices.

The topic guide (see Table 1) included views and experiences of the HPV vaccine and evaluation of publicly available information and media reports (Centers for Disease Control and Protection [CDC], 2015; Jo’s Cervical Cancer Trust, 2014). Participants were offered a £20 gift card to compensate for travel, time and childcare costs. All 11 groups (focus groups/paired interviews) were facilitated by EC and in most cases, another member of the research team.

**Table 1.** Topic guide.

• *Discussion prior to stimulus material* ○ Views and experiences of the HPV vaccine ○ Knowledge about HPV and the vaccine, including personal attitudes towards vaccination ○ Awareness of the vaccine among family/friends/community ○ Views on who should receive the vaccine ○ Sources of information about HPV and the vaccine
• *Discussion after stimulus material – health information leaflets/media reports* ○ Views on information made available and its suitability within communities ○ Understandings of HPV and the vaccine based on the information provided ○ Views on who should get the vaccine and implementation strategies ○ Suggestions for improving information on HPV and the vaccine ○ Barriers and enablers to increasing vaccine uptake within communities.

## Analysis

Focus groups and paired interviews were audio-recorded, transcribed verbatim and anonymised, and participants were given pseudonyms. The data sets did not differ substantially, so they were analysed as one corpus data set. To enhance credibility and rigour, two of the nine members of the research team coded each transcript for recurring topics and themes, discourses and counter-discourses (Starks and Trinidad, 2007), signified through language use, with a focus on participant discussions around barriers, solutions and strategies to increase knowledge, acceptability and access to the vaccine. Field notes and analytic summaries were discussed with other team members at monthly meetings. As a final step in the analysis, researchers explored participant solutions within the data and displayed patterns and relationships as a model. Cultural and contextual enablers to decision-making are central tenets traversing four key strategies: information-giving, vaccine provision, education across generations and modes of communication. In keeping with our analytic approach, findings reported here refer to young people’s accounts and perceptions of the HPV vaccine in Scotland.

## Findings

Participants initially revealed minimal understanding of HPV or the purpose of the vaccine, despite acknowledging that information was provided around the time of vaccination. Accounts suggested that sex education had failed to include information about HPV and there were few, if any, opportunities to discuss or learn about HPV and/or related cancers at school or elsewhere. Most were unaware how HPV affected men and were surprised to learn that other countries offer this vaccination to boys/young men. These discussions prompted participants to provide ideas about how HPV education could be delivered in a more impactful way.

Similarly, after HPV educational information was provided, participants were surprised regarding the magnitude of the virus. Many were confused by the different types of the virus, its links with cancer and how that develops, and they voiced concern about its prevalence and obscurity. In contrast, some expressed little or no anxieties about HPV, citing religious grounds, suggesting a distancing from the virus by those with specific moral positions. Several Muslim women, aged less than 20, questioned the implications of the virus for them if they were celibate and intending a monogamous life after marriage. Young Sikh men expressed surprise about men not being offered the vaccine and highlighted barriers to discussing HPV transmission routes openly within their community. Similarly, young Muslim men discussed how HPV challenges cultural or religious norms which arouse parental fears of promiscuity, which in turn led to parental disapproval of the vaccine and potential stigmatisation of families who accept vaccination. Accounts of participants of Indian/Pakistan descent and/or Muslim/Sikh religion were contested by men and women of African descent who felt they could talk relatively about sexual practices from a young age. These discussions prompted participants to talk about solutions to overcoming intergenerational concerns and culturally sensitive strategies for different ‘ages and stages’, genders and settings.

### Constructing solutions for the HPV vaccine

**Figure 1.**
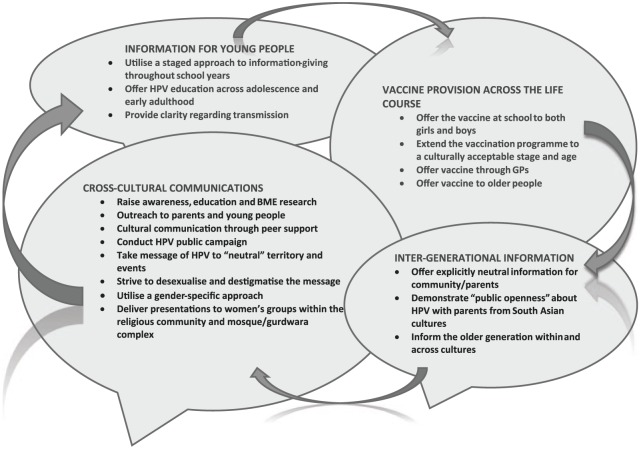
Cross-cultural community engagement model for HPV.

Having identified cultural barriers, participants discussed how practitioners and policymakers could raise the profile of HPV across diverse communities. These factors and processes are illustrated within the community engagement model (Figure 1) and following discussion.

### Providing targeted and flexible information for young people

Relationships between sexual activity, transmission and cancer were largely unclear to participants and discussions centred on whether practitioners should emphasise cancer or sexually transmitted infections. A staged approach was offered as a solution – delivering information at age-appropriate and differing developmental stages during school and extending this across the lifespan – for example, in contexts such as further education institutions and primary care. These settings were deemed to be ‘neutral’ contexts where cultural sensitivities or conflicts could be avoided and where cultural norms (i.e. marriage) could be enablers. These would offer additional opportunities for young people from minority ethnic communities to consider receiving the vaccine, if they had previously refused or missed the opportunity as adolescents:

Amberash: Maybe later on … sex education isn’t for, like, 12, 13 year olds. I think it’s more older, that’s when they can introduce it and make the connection.

Chidimma: I don’t think I would have processed the information at that time, even at 16. (Group 5: Black/African men, aged 24–26)

Priya: I think when you’re that age [9–14 years] … I’d want to know the general information but I wouldn’t want to go into such detail because I wouldn’t be that interested. (Group 7: Women of South Asian and West Indian descent, aged 24–25)

Miriam: In the Muslim community, round about the 20s is when most people begin to get married … I think that’s a good age to actually raise awareness of it. (Group 4: Asian/Muslim women, aged 22–23)

In relation to health literacy, participants commented on a contradiction within public health provision – the scale of infection and yet the lack of accessible information. They offered notions of what might be concise and clear information since HPV appeared to challenge commonly accepted HIV-related safer sex messages (NHS Scotland, 2014). Participants underlined a public health challenge – how to achieve a balance between raising sexual health awareness versus arousing young people’s fears:

Faiza: Quite a lot of people at my school didn’t get it because we didn’t actually know how important it was … we didn’t really know … if it was, like, vital.

Basima: It’s because we never really went into depth about why we need it. (Group 8: Arab/Muslim women, aged 16–17)

Ayotunde: I think they are trying to make it look, like, worse than it is … because some of them … are cured by immune system. They don’t need to tell us, it’s a hundred [different genotypes].

Chinaka: They don’t need to arouse the fear in people. (Group 1: Black/African women, aged 16–23)

A flexible age-appropriate and culturally sensitive approach, offering targeted vaccine information, was proposed by participants. Medical and public health practitioners could provide a range of information opportunities across the life-course to raise awareness, regardless of current boundaries of a school-based, gender-specific programme.

### Vaccine provision across the life-course

Providing the vaccine within a variety of contexts was deemed necessary in order to offer more choice and privacy to young people when considering HPV information and deciding whether to receive the vaccination. Participants favoured continuing the current school’s vaccination programme with the inclusion of boys, but widening the programme to offer the vaccine in colleges and primary care. Thus, they advocated a flexible, staged approach, extending the vaccination programme to people older than are currently included. The use of trusted sources (e.g. General Practitioners) seemed important, acknowledging young adolescents’ engagement in sexual activity in general, and more specifically one that reflects their age of sexual debut around the time of marriage in order to be culturally congruent:

Sukhbir: If they get it when they’re 12, they’re just going to be like, oh yes, I got a jag [injection] today, but I don’t know what it was for. But, if they get it later on it will like affect their lifestyle in a way.

Achint: Just reiterate it’s not just girls can get it as well, guys can get it, [and] … if they’re not going to give vaccine to boys at least tell them about what HPV is, not have exclusive talks with girls. (Group 10: Indian/Sikh men, aged 16–23)

Mandeep: For me, it would be the later years, even as late as university … If someone offered me the HPV vaccination right now, it might be no. Why do I need another injection for something which I personally deem as probably very low risk to me. (Group 6: Asian/Muslim men, aged 23)

Miriam: Because in the Asian community, people need it later, when they’re in their 20s, mid-20s. Say the vaccine and testing is available from your GP if they want to get it. Just so they know they haven’t missed their opportunity. (Group 4: Asian/Muslim women, aged 22–23)

Regardless of the religious persuasion, young women were particularly concerned to discover that men are at risk of developing HPV-related cancers and transmitting the virus to others. Repeated questioning about routes of transmission and pleas for equality – both sexes to be vaccinated – suggested some underlying anxiety about perceived risk of acquiring HPV:

Chinaka: And then the older guys are still sexually active. What about them? It should just be mandatory like tax.

Ebele: Any guy should be able to get it, whether they’re 60 or they’re 13. (Group 1: Black/African women, aged 16–23)

Public health policy was frequently problematised, especially regarding how HPV information and the vaccine are delivered inside or outside a school programme where mass vaccinations are administered within a defined immunisation schedule. Having considered the delivery of the immunisation programme in secular contexts such as schools and health centres, participants discussed additional strategies to address tensions within their own cultural contexts.

### The need for intergenerational information

Social norms within families and communities and places of worship were emphasised. Young men from South Asian communities positioned themselves as powerless to resolve intergenerational tensions regarding ‘acceptable’ or open dialogue around HPV. In contrast, the women suggested various opportunities to promote intergenerational information. Across all cultural groups, participants advocated ongoing education for the older generation:

Amitt: The older generation, culturally don’t like to talk about how it is. They like to talk about how it should be … Depends on where you put this information. If you put it in universities … you wouldn’t be as wary of cultural effects, but if you’re trying to target a mosque or something like that, be very cautious of the way you approach it. (Group 6: Asian/Muslim men, aged 23)

Aminah: It’s like a youth group, but for elderly women, so look for groups like that, or other women’s classes … I think they would be more comfortable … and keener to listen … because they might feel a bit embarrassed, and then they can ask questions. (Group 4: Asian/Muslim women, aged 22–23)

To avoid alienating parents, Muslim men suggested greater transparency and a more personalised approach, for example, by offering parents different ways to contact the school about the vaccine:

Khaliq: School newsletters is a good way, put up a little section explaining it, and offer them a contact line or an email, … to enquire about or if you’re worried about HPV just phone this [number], or come to school, or talk to us. (Group 9: Arab/Muslim men, aged 19–20)

In comparison, respondents in a group of Black men argued for a broader approach, educating everyone without fear of repercussions:

Amberash: I think parents as well need to be educated about this, so if kids are educated in school, parents have first-hand information already. What I want to see is some leaflets distributed in health centres as well talking about this, which is similar to HIV. (Group 5: Black/African men, aged 24–26)

As a whole, participants required targeted education to address intergenerational concerns and HPV promotion strategies delivered across a range of settings. Notably, they recommended different modes of communication across South Asian and Black African cultures (Kandula et al., 2015).

### Cross-cultural communication

Finally, participants called for a broad, diverse and comprehensive approach to tackle the dearth of public awareness about HPV within their communities. This included community outreach for public health practitioners to contact and work at a local and national level; exploiting opportunities such as local community and public events; delivering information in a sensitive manner engendering discussion, trust and dialogue; striving to desexualise and destigmatise the message; and taking it to gender-specific arenas such as established women’s groups in religious or cultural centres (Resnicow et al., 1999). Communicating through community members and leaders was thought to be paramount. A salient message was the necessity for cultural and physical proximity – communities need to see signs and action from government to ‘work with us’ and ‘through us’:

Amberash: The community, government, realised they find it difficult to deal with us, so we decided to create our own organisation. Now they do approach us and find ways and answers from us … Just train somebody who has more knowledge, who has ideas of minorities, approach more people from minorities, have seminars so people can put their view forward. (Group 5: Black/African men, aged 24–26)

Participants across all communities suggested a public campaign about HPV. This suited some men and women from South Asian communities who preferred messages to be delivered in ‘neutral’ territory:

Mandeep: There are definitely Muslims with alcohol problems, or problems related with STIs … but in a mosque environment, it’s very hard to get them to admit it. That would be like one of the last places they would go to look for information. In an educational environment, in school or universities, student union, all of those kind of places are I think much better suited. (Group 6: Asian/Muslim men, aged 23)

Layla: I wouldn’t hand it out in a mosque, no.

Faiza: I think they might find that a bit … offensive, yeah. I think they should stick to high schools. (Group 8: Arab/Muslim women, aged 16–17)

Mandeep’s account acknowledges ‘Muslims with alcohol problems, or problems related with STIs’, as a hidden group within cultural norms and expectations. This points to tensions around those individuals who are at risk of HPV through covert sexual practices versus those individuals who are protected from risk through cultural expectations around marriage as sexual debut. Inherent within the account is the precarious notion of deeming marriage as protective for sexual health within such communities.

Men from the Gurdwara suggested that peer support from within their community was preferable to relying on outsiders to interpret and explain the vaccine. They warned aligning it with HIV would stigmatise the virus within their community. Muslim young men also raised the issue of stigma if the vaccine was associated with men who have sex with men:

Ramindar: I don’t know if it’s as bad as HIV though, that might kind of confuse things. HIV was regarded as a big taboo, it still kind of is. If someone had HIV or someone mentioned it, it was something which caused a divide, and it might be, by aligning it with that you kind of … (Group 10: Indian/Sikh men, aged 16–23)

Mandeep: If you’re aiming it at a specific, like a Muslim culture, it would be more receptive if it was sold almost like the common cold where anyone can get it through physical contact, but you’re more likely to get it if you have sex.

Kumar: [the] consequence of rolling it out to gay men first … it becomes something seen as, oh this is important for gay men. So it almost gets, not stigma, but … from a cultural perspective, we might tend to say, that’s only important for gay men, so it’s not really important. (Group 6: Asian/Muslim men, aged 23)

In contrast to this fear of offending elders or causing disrespect, some women asked for presentations to be delivered to women’s groups within the religious community and mosque/Gurdwara complexes:

Deepti: I think we should start something in the Gurdwara itself for the younger kids that come here.

Gurpreet: Or just for women, like younger women …They don’t want to talk about the same subject with men.

Divya: If they’re not comfortable speaking about it maybe we can have like wee closed groups like this. (Group 11: Indian/Sikh women, aged 17–20)

## Discussion

An HPV community engagement model, derived from our analysis, conceptualises a process of engagement and empowerment recognising context and complexity as a way forward for policy and practice. Participants provided strategies for tailored public health interventions – how they could be delivered, by whom, when and where. The model embraces cultural sensitivities and diversity with the purpose of raising acceptability of the HPV vaccine and promoting self-determinism. Differing participant views shed light on the question of whether HPV messages should be gendered, culturally specific, general or neutral. As indicated, the context of delivery will determine specificity and tone of the information – each mode requires negotiation with a discrete community and is important for informed decision-making for the vaccine.

Participants called for accessible, interactive, experiential and memorable information. This is crucial from a policy perspective because in Scotland, from 1 June 2016, the age of first cervical smear will be offered at 25 years of age – 14 years after HPV vaccination – leading to a potential ‘disconnect’ between HPV, cancer and screening. Some educational interventions show promising results in raising awareness of cancer and healthy lifestyles (Sherman et al., 2015). One intervention comprising a 60-minute lecture followed by a 30-minute video led to increased awareness of risk factors, prevention strategies and warning signs for cancer in undergraduate students (Hwang, 2013). As study participants illustrated, delivering HPV education and vaccination within schools could bypass cultural barriers; however, an active role in the school educational programme would be essential (Cooper et al., 2016).

White Northern hemisphere norms and assumptions about age of sexual debut were challenged by our participants but left some ‘hidden’ individuals vulnerable within cultural expectations. This indicates a need for transparency regarding immunological reasoning and rationale behind current age of vaccination as well as inviting communities to attend at culturally appropriate stages, through to early adulthood. This issue was echoed by a survey of female family practitioners in Pakistan (*n* = 100) where 39% believed the appropriate age for receiving the vaccine was between 25 and 30 years (Sanaullah et al., 2012).

Furthermore, our findings strongly suggest that intergenerational issues need attention. Knowledge transfer may be influenced by how older people interpret HPV information or act as gatekeepers for the community. Participants raised the possibility of consulting directly with elders about HPV during future initiatives. Targeting the vaccine towards men who have sex with men could sexualise the vaccine and lead to further misunderstandings, as highlighted by our Muslim participants. Working in partnership with communities will be essential if policymakers are to devise appropriate non-stigmatising risk communication messages – for example, how much information ought to be disclosed taking into account HPV risk factors, morbidity and mortality rates (Cunningham-Erves and Talbott, 2015). A trial conducted in Hong Kong demonstrated that HPV messages containing specific destigmatising components could reduce public stigma towards HPV (Kwan et al., 2010).

Participant accounts revealed a tension between the need for social integration and localised or ‘neutral’ information for specific communities. The cross-cultural model provides a tool for practitioners and policymakers to engage with community fora, grassroots organisations and advocacy organisations. It stresses the importance of working with people who are in proximity both physically and culturally to discrete communities. A community-engaged research process was described by Katz and Paskett (2015). Comparing the interactions and perspectives of members from two different cultures, they concluded that both shared the same goal – to keep their child healthy – but their views varied regarding content and format of the interventions emphasising the need for context-specific interventions. A flexible format allowed researchers to tailor the programme based on a child’s gender, age, race, and ethnicity.

The apparent need for health information to be made available in a variety of formats and settings was mirrored in a US study of mothers and adolescents from Somali, Eritrean and Hispanic communities (Greenfield et al., 2015). All three ethnic groups expressed a desire to access vaccine information in their respective language in varied formats, and within community classes and small group settings, mainly because many were not literate in English. Hispanic parents were receptive to information via health fairs, schools and radio, whereas Eritrean parents preferred information translated through doctors and television. The authors conclude immunisation outreach should be conducted in specific culturally appropriate ways. This implies a community engagement process that involves community participation, intersectoral collaboration and equity with an emphasis on self-reliance and determination. Age of vaccination and where to vaccinate could be customised through negotiation with discrete communities.

A key strength of our proposed model is that it comprises insights from in-depth discussions with several minority ethnic communities and therefore contributes to a neglected area in research, policy and practice. Participants signalled four areas for practitioners and policymakers to focus on: a targeted information strategy, an extended and flexible gender-neutral vaccination programme, proactive culturally sensitive education across all age groups and partnership working to adopt guidance from each community.

### Limitations of the study

We acknowledge that this is a tentative model which will require further testing and refinement with more diverse cultural groups and within different cultural settings, as well as parents, carers and other family members. This study involved a relatively small sample of young people from three geographical regions in Scotland, whose demographic and ethnic backgrounds were varied. Our sample cannot be considered representative of the wider population of young people from all ethnic groups in Scotland or elsewhere. Although our findings are not generalisable, age, gender and generational status were explored and the model and its processes may be transferrable to other contexts. These and other key influences, such as socio-economic status, are likely to shape perceptions and intentions. An understanding of these intersectional influences within different ethnic groups is essential to inform strategies which promote vaccination uptake. Finally, the pragmatics of these suggestions need to be further explored along with the effectiveness in reaching targeted audiences.

## Conclusion

This paper illustrates a novel HPV community engagement model, inductively constructed from the accounts of young people from Black, Asian and other minority ethnic communities. Implementation of the model could enable dialogue with diverse communities, combining a bottom-up with a top-down approach where priorities can be set by policymakers with the delivery guided by the needs of each specific community. Tackling diversity in this way could increase the profile and future uptake of HPV vaccines.
